# Editorial: Pulmonary Hypertension in the Modern Era: Science and Clinical Practice

**DOI:** 10.3389/fmed.2021.785181

**Published:** 2021-10-28

**Authors:** Elena A. Goncharova, Harm J. Bogaard, Vinicio A. de Jesus Perez

**Affiliations:** ^1^Division of Pulmonary, Critical Care and Sleep Medicine, Department of Internal Medicine, Lung Center, School of Medicine, University of California, Davis, Davis, CA, United States; ^2^Department of Pulmonary Medicine, Amsterdam University Medical Center, Amsterdam, Netherlands; ^3^Division of Pulmonary, Allergy and Critical Care Medicine, Stanford University Medical Center, Stanford, CA, United States

**Keywords:** pulmonary hypertension, group 1 PH, group 2 PH, group 5 PH, genetics and genomics, endothelial dysfunction

Pulmonary hypertension (PH) comprises five groups of progressive cardiopulmonary diseases defined as mean pulmonary arterial (PA) pressure (mPAP) ≥ 20 mmHg (1), leading to right heart failure and premature death. Despite past advances, the pathobiology of different groups of PH is incompletely understood, and diagnostic and treatment options are limited.

Pulmonary arterial hypertension (PAH, Group I PH) is a rare group of conditions that includes familiar and idiopathic forms. Multiple genetic and genomic risk factors have been identified (2), but much remains to discover. Using whole genome sequencing of two unrelated families with PAH, Pienkos et al. identified two novel rare variants in *TNIP2* and *TRAF2*, which could promote an altered immune response, pulmonary vascular remodeling and PH by permitting overactivation of NF-κB. In parallel studies, Rathinasabapathy et al. created transgenic mice harboring the human *Cav1* mutation. These researchers found that the *Cav1* mutation results in metabolic deficiencies, development of mild PH, reduced spontaneous exercise, and an inflammatory phenotype. This study adds to emerging evidence suggesting a role for insulin deficiency and exercise intolerance, and further supports the role of inflammation in PAH.

Other critical players in PAH are PA endothelial cells (PAECs), and the mechanisms by which PAECs regulate immune function and inflammation in PAH continue to attract attention. In this issue of the Journal, Bhagwani et al. provide a comprehensive review of the role of toll-like receptors, central players in innate immunity, in the immune and non-immune functions of endothelial cells in PH. One important but under-used tool in PAH pre-clinical research is high-resolution imaging, which was successfully used to study pathobiology of other diseases (3). In this special issue, Klouda et al. combined super-resolution microscopy with precision-cut lung slices and clearing technology to develop a novel evolutionary method to study cell behavior *ex vivo*, trace and reconstruct pulmonary vasculature, and address fundamental questions relevant to a wide variety of vascular disorders including PAH.

In addition to idiopathic and familiar forms, group 1 PH includes portopulmonary hypertension (PoPH) that afflicts ~6–8% of patients with portal hypertension and is associated with high morbidity and mortality, particularly in the context of liver transplantation. In this issue, Thomas et al. review the most recent advances in our understanding of the pathogenesis and clinical course of PoPH. The last decade has seen exciting developments in the management of PoPH, with large clinical trials demonstrating the efficacy of several PH-specific therapies in improving hemodynamics and clinical outcomes. While there is more work to be done, the authors' state-of-the-art summary leaves us enthusiastic for future progress in the field of PoPH research.

As new oral prostacyclin therapies and prostacyclin agonists have become available for the treatment of PAH, there is an increasing need for methods for transitioning between oral, inhaled and parenteral formulations. Pan et al. describe their experience of successful transitioning between different prostanoid therapies. Particularly because a standardized approach in transitioning safely between different formulations without compromising treatment efficacy is lacking, their case series gives valuable insight into sensible clinical practice and the pharmacokinetics of various prostanoid formulations. In a parallel report, Kopp et al. share their experience with one particular method of prostanoid therapy: intravenous treprostenil administration *via* a Lenus Pro® Implantable Pump. While this method has clear advantages, the authors witnessed unexpected acceleration in treprostinil delivery in two patients. The flow rate delivered by the pumps in these 2 patients progressively exceeded the manufacturer admitted margin of error with increases of more than 50% of the expected flow rate at the end of follow up. Spontaneous flow increase from such an implantable pump is a potentially major pitfall, which needs to be identified and actively managed by the responsible clinicians. Prospective studies are needed to assess the long-term safety and efficacy of treprostinil administration by this device.

One of the most common and lethal forms of PH is PH due to left heart disease (PH-LHD, group 2 PH) (4). In their review article, Todd and Lai discuss the challenges and opportunities in developing biomarkers for diagnosing and managing PH-LHD. Presently, the only biomarkers available are B-type natriuretic peptide and N-terminal proBNP; however, data for these biomarkers comes mainly from studies in non-PH-LHD patients, which may not necessarily capture the complex phenotypes of PH-LHD patients. In their paper, the authors discuss exciting studies pointing at endothelin-1, vascular endothelial growth factor-D, and microRNA-206 as new potential circulating biomarkers for patients with PH-LHD and how they could serve as the basis of future risk stratification tools for making management decisions in PH-LHD patients.

A significant challenge for improving the quality of life and survival of PH-LHD patients is the lack of effective treatments. To date, there is no definite evidence that PH-specific therapies can be routinely used to treat patients with PH-CHD. Given this problem, there is an active interest in investigating experimental treatments that could target mechanisms inherent to the pathogenesis of PH-LHD. In this special issue, Mulkareddy and Simon provide a comprehensive review of the evidence that supports metformin as a prime candidate for clinical trials in PH-LHD. Given the strong association between metabolic dysregulation, insulin resistance, and cardiovascular diseases, investigators have identified the anti-diabetic drug metformin as a potential treatment for PH-LHD. Besides counteracting insulin resistance in type 2 diabetes mellitus, metformin has a range of beneficial effects that could potentially halt the progression of cardiopulmonary remodeling in PH-LHD and improve cardiac function, a major source of morbidity and mortality in these patients. Repurposing metformin for PH-LHD also offers the advantage of improving access to a potentially life-saving drug since the availability of generic formulations could help make this drug affordable and more accessible to patients around the world.

Group 5 PH comprises heterogeneous group of PH secondary to multifactorial mechanisms.

Al-Qadi et al. provide a thorough review of the epidemiology, pathogenesis and clinical approach of group 5 PH, with a specific focus on hematologic disorders, sarcoidosis and chronic renal failure. These forms of PH are caused by a mix of pathophysiologic drivers and can present with precapillary as well as postcapillary disease. The pathogenesis of most forms of group 5 PH includes hypercoagulability, altered vasomotor tone and varied degrees of cardiomyopathy due to a chronic high flow state or intrinsic involvement of the myocardium. In addition, specific to sarcoidosis are advanced interstitial lung disease, granulomatous obliteration of arterioles or venules and extrinsic compression of pulmonary arteries (and venous stenosis) by enlarged lymph nodes or fibrosing mediastinitis. Cardiotoxic and pulmonary toxic effects of medication deserve mention too, particularly the use of tyrosine kinase and alkylating agents in myeoloproliferative disorders. Management of group 5 PH primarily involves optimizing treatment of the underlying disease and general measures such as supplemental oxygen, cautiously used diuretics and anticoagulation. PH-targeted therapy is usually reserved for patients with moderate to severe (based on functional class) RHC-confirmed pre-capillary PH with relatively reduced cardiac output.

In conclusion, this special issue highlights continuous interest in pulmonary hypertension research, spanning genetics and genomics, molecular mechanisms of PH pathogenesis, novel imaging techniques, and clinical studies. Together, efforts of many researchers continue to improve our understanding of disease pathobiology and treatment options for patients with PH.

## Author Contributions

EG, HB, and VJ drafted and edited the manuscript. All authors contributed to the article and approved the submitted version.

## Conflict of Interest

The authors declare that the research was conducted in the absence of any commercial or financial relationships that could be construed as a potential conflict of interest.

## Publisher's Note

All claims expressed in this article are solely those of the authors and do not necessarily represent those of their affiliated organizations, or those of the publisher, the editors and the reviewers. Any product that may be evaluated in this article, or claim that may be made by its manufacturer, is not guaranteed or endorsed by the publisher.
